# Beyond radicalization: the 3N model and its application to criminal attitudes in high-risk contexts

**DOI:** 10.3389/fpsyg.2025.1498936

**Published:** 2025-02-17

**Authors:** Jocelyn J. Bélanger, Michael Wolfowicz, Hayat Mohammad, Roberto M. Lobato, Michelle Blaya Burgo, Laura Rico-Bustamante, José M. Martín-Criado, Manuel Moyano

**Affiliations:** ^1^Department of Arts and Sciences, Carnegie Mellon University in Qatar, Doha, Qatar; ^2^Institute of Criminology, Hebrew University of Jerusalem, Jerusalem, Israel; ^3^Department of Psychology, University of Peshawar, Peshawar, Pakistan; ^4^Departamento de Ciencias de la Salud, Universidad de Burgos, Burgos, Spain; ^5^Department of Psychology, Claremont Graduate University, Claremont, CA, United States; ^6^Department of Psychology, University of Córdoba, Córdoba, Spain

**Keywords:** 3N model, search for meaning, deviant associations, criminal attitudes, radicalization

## Abstract

**Introduction:**

This research integrates criminological and psychological literature by applying the 3N model of radicalization to predict criminal attitudes. Specifically, we conceptualize “need” as the search for meaning, “networks” as deviant associations, and “narrative” as criminal attitudes. We examine the roles of these factors across diverse cultural contexts and investigate the effectiveness of prosocial models in redirecting the search for meaning away from criminal attitudes.

**Methods:**

Three studies were conducted to examine the relationships between the search for meaning, deviant associations, and criminal attitudes. Study 1A sampled former inmates in Pakistan (*N* = 243), while Study 1B (*N* = 402) and Study 2 (*N* = 330) focused on at-risk youth in southern Spain. Study 2 further tested a field intervention using positive role models to mitigate the search for meaning and criminal attitudes.

**Results:**

Findings from Studies 1A and 1B indicate a significant indirect effect of the search for meaning on criminal attitudes, mediated through deviant associations. Additionally, Study 2 demonstrates that exposure to prosocial role models reduces both the search for meaning and criminal attitudes, supporting the effectiveness of intervention strategies.

**Discussion:**

These findings highlight the utility of the 3N model in understanding criminal attitudes within diverse high-risk contexts. By bridging psychological and criminological perspectives, this research offers a framework for prevention and intervention strategies targeting individuals vulnerable to criminal influences.

## Introduction

Criminology and psychology both share a common interest in comprehending criminality, albeit through distinct paths. While criminology has primarily focused on social and structural factors, psychology has shown greater interest in cognitive elements (Hollin, 1989). Notwithstanding their differences, there are several areas of convergence between these disciplines, with criminal attitudes arguably being the most significant point of intersection. These attitudes involve positive evaluations of criminal behaviors and have been identified as influential predictors of actual criminal conduct (Helmus et al., 2013).

Interestingly, there is a conceptual overlap between criminal attitudes and radical attitudes. The latter pertains to positive evaluations of radical behaviors, including illegal acts and violence, driven by a cause or ideology (McCauley and Moskalenko, 2017).^1^ Notably, both criminal and radical cognitions involve permissive attitudes toward law-breaking, especially violence, and research demonstrates a strong consistency between attitudes and behaviors in both domains (Wolfowicz et al., 2020, 2021). Furthermore, recent systematic reviews and meta-analyses have revealed that both radical and criminal outcomes are predicted by similar risk and protective factors. These factors include familiar criminogenic elements such as criminal history, low self-control, and deviant peers (Lösel et al., 2018). Additionally, there is evidence of strong correlations between radical attitudes and other risk factors that have hereto not been investigated with respect to criminal attitudes, particularly the search for meaning, also known as the quest for personal significance (Wolfowicz et al., 2020, 2021).

Building upon prior work, this research aims to advance the integration of criminological and psychological literature by applying the 3N model of radicalization (Kruglanski et al., 2014; Kruglanski et al., 2019) to the study of criminal attitudes. This model encompasses the domains of needs, networks, and narratives. By utilizing this model, we investigate the role of significance seeking—a universal human *need* associated with radical attitudes—and its connection with deviant associations (*networks*) to deepen our understanding of the adoption of criminal attitudes (*narratives*).

### Needs

Human beings are intrinsically motivated by innate needs that profoundly influence a wide array of cognitions, decisions, and behaviors. In criminology, the concept of “need” is closely associated with the notion of low self-control (Gottfredson and Hirschi, 1990), while psychology highlights its sub-components, namely, impulsivity and thrill-seeking (Steinberg et al., 2008). Within this context, the urge to seek immediate gratification is viewed as a propensity that drives criminality (Gottfredson and Hirschi, 1990). According to a recent meta-analysis, these need-related factors (e.g., self-control, thrill-seeking) rank among the most significant predictors of radicalization outcomes, encompassing both cognitive aspects (e.g., radical attitudes) and involvement in radical behaviors, such as perpetrating violence against others in the name of a cause (Wolfowicz et al., 2020).

However, the expanding literature on radicalization has uncovered other needs that may impact criminal attitudes. Among these is the universal need for significance, which encompasses the desire to be someone and to matter (Kruglanski et al., 2019). When individuals experience a loss of significance due to life events such as personal failure or social alienation, they naturally seek ways to restore this loss (Bélanger et al., 2019). In situations where individuals cannot regain their significance through legitimate means, they may turn to seeking restoration through retaliatory actions against entities they hold responsible for the loss of personal meaning. In line with this view, numerous studies have found robust associations between the experience of a loss of significance and the presence of radical attitudes (Dugas et al., 2016; Wolfowicz et al., 2021).

The need for significance is also evident in criminological explanations of deviance. For instance, subculture theories emphasize the pursuit of honor and status as primary motivators for engaging in violence and gang-related crime (Goldstein and Soriano, 1994). The idea that the absence of personal significance may contribute to the adoption of deviant attitudes also finds support in Frankl’s (1997) work. According to Frankl, when individuals’ “will to meaning” cannot be fulfilled, they may seek to fill the void with a “will to pleasure,” leading to addiction, or a “will to power,” resulting in violence.

Starting in the late 1990s, the concept of meaning in life and logotherapy, Frankl’s therapeutic method, found application within prison-based interventions aimed at reducing the likelihood of recidivism. However, few studies have directly tested the effects of these interventions on meaning in life itself. Instead, many of these programs tend to focus on spirituality, which combines factors such as life meaning, purpose, and others. One such example is the Good Lives Model (GLM) of rehabilitation, which operates on the belief that criminality arises from an inability to attain a meaningful life through normative or pro-social means due to a lack of competencies. GLM programs concentrate on developing these competencies and fostering pro-social networks, enabling individuals to achieve a meaningful and “good life.” Studies show that GLM models including group-based therapies (networks) show improvements in meaning in life (needs) and reduced criminal attitudes (narratives), reducing the likelihood of recidivism (Harkins et al., 2012).

However, as this body of evidence shows, research has primarily focused on examining the presence of meaning in life, while giving comparatively less attention to the search for meaning, which is a distinct aspect (Steger et al., 2006). Furthermore, the majority of research has focused on offenders and has measured significance after the act of offending. Thus, the relationship between the search for meaning and criminal attitudes, including its relevance to the risk of offending, remains unexplored. Based on evidence pertaining to radical attitudes and the 3N model, we hypothesize that the search of meaning may increase the likelihood of adopting criminal attitudes, because of its influence on deviant associations. This relationship is discussed in the next section.

### Networks

Criminologists and psychologists recognize the influence of social connections on shaping deviant attitudes, with deviant peers consistently identified as a risk factor for both radical (Wolfowicz et al., 2020) and criminal attitudes (Pratt et al., 2010). Moreover, deviant associations have been found to mediate the relationship between various individual-level risk factors, such as needs and deviant attitudes. For instance, Walters (2017) showed that the impact of thrill-seeking on criminal attitudes is largely mediated by deviant associations. These findings align closely with the radicalization literature. Indeed, research based on the 3N model suggests that the search for personal significance redirects attention toward restoring it, and one way for restoration involves identifying with a group (Kruglanski et al., 2019). If the means of restoring meaning identified within the reference group involve political violence, individuals may be drawn to use those means. This suggests that reducing involvement with deviant associations or promoting pro-social connections may redirect the search for meaning in positive directions (Schumpe et al., 2018). In the present research, we test such a hypothesis, examining not only the direct effects of deviant associations on criminal attitudes, but also their role as a mediator between people’s search for meaning and criminal attitudes. We now turn to the concept of narratives in the criminological and psychological literature and explore how to measure these narratives in a manner that enhances the integration of both perspectives.

### Narratives

Engaging in criminal behaviors typically hinges on the offender holding favorable attitudes toward the specific behavior or justifying it (Akers, 1998). Similarly, radical behaviors are strongly predicted by radical attitudes, which involve positive evaluations of violence to further a cause (Wolfowicz et al., 2020). As previously mentioned, beliefs about the legitimacy of violence are often deeply embedded in the group the individual belongs to such as a criminal gang or a terrorist group. Within these groups, other members validate and reinforce the narrative, rewarding individuals with respect and admiration for adhering to its principles (Kruglanski et al., 2019).

While radicalization research has largely concentrated on attitudinal outcomes, resulting in the development of diverse tools to measure and support radical attitudes, criminology has primarily prioritized behavioral outcomes. Nevertheless, several measures of criminal cognition have been developed, such as the Psychological Inventory of Criminal Thinking Styles and the Measure of Criminal Attitudes and Associates scales. Although these validated instruments and their subscales have proven highly beneficial, they often blend various cognitions with attitudes. These cognitive components frequently encompass thinking styles like impulsivity or moral disengagement, while attitudes primarily refer to evaluations or orientations toward offending behaviors.

## Overview

This research examines the core concepts of the 3N model of radicalization to predict criminal attitudes across three distinct contexts. A key objective is to address the measurement challenges associated with criminal attitudes by employing an adapted version of a radical attitude scale from Bélanger et al. (2019). This modified scale allows us to demonstrate the overlap between radical and criminal attitudes and provides insight into how criminal attitudes (or narratives) emerge as a result of needs and networks.

Building on prior research, we emphasize the sequence of predictors beginning with the search for meaning, progressing to deviant associations, and ultimately influencing criminal attitudes. While the 3N model allows for various temporal orders among needs, narratives, and networks (Kruglanski et al., 2019), a specific trajectory—search for meaning leading to radical attitudes via deviant associations—has been documented across diverse ideological samples and research designs (correlational and experimental) (Dugas et al., 2016; Bélanger et al., 2020; Lobato et al., 2021). Our objective is to replicate this model within the context of criminal attitudes.

Study 1A focused on former inmates in Pakistan, while Study 1B examined a non-criminal but high-risk youth population in Spain. Based on the theoretical framework, we hypothesize that H1: the relationship between the search for meaning and criminal attitudes is mediated by deviant associations. Study 2 extends the model’s application to an intervention conducted in schools within deprived areas of southern Spain, where we hypothesize that H2: promoting pro-social associations can redirect the search for meaning away from criminal attitudes. Ethical approval for this study was obtained from the Institutional Review Board under protocol number [masked for review].

## General methods

This section outlines the shared methodological approaches applied across all three studies, emphasizing the steps taken to ensure consistency, cultural relevance, and methodological rigor. Given the diversity of the study contexts—spanning different cultural, linguistic, and educational backgrounds—it was essential to adapt instruments appropriately while maintaining their validity and reliability.

### Instrument adaptation and item selection

Items were translated into Urdu (Study 1A) and Spanish (Studies 1B and 2), then back-translated into English by independent bilingual researchers to ensure equivalence. Discrepancies were resolved through consensus, with input from local experts and educators to ensure clarity and contextual relevance.

The selection and number of items varied across studies based on pilot testing, which identified the most reliable and contextually appropriate items for each population. Less effective items were excluded, and brief measures were prioritized to minimize participant burden, especially in contexts with low literacy or time constraints. These adaptations ensured the instruments remained meaningful, accessible, and consistent with the constructs being measured.

Qualitative feedback from teachers and community advisors further ensured the instruments resonated with participants’ experiences. Special attention was given to balancing cultural adaptation with theoretical and psychometric rigor, ensuring the instruments were valid and reliable. This iterative refinement process enabled meaningful cross-study comparisons while addressing the practical challenges of working with vulnerable populations.

Furthermore, the Bélanger et al. (2019) scale, originally designed to measure support for political violence in the context of violent extremism, was adapted in this research to assess criminal attitudes, specifically focusing on non-ideological violence. This adaptation aligns with the study’s aim to examine attitudes toward crime rather than ideologically motivated actions. The most relevant items were selected based on both qualitative and quantitative insights from prior field research with similar samples, ensuring the scale’s applicability to the context of criminal behavior.

### Data preparation and analysis plan

To ensure methodological rigor and clarity, the data preparation and analysis steps were consistent across all studies:

Bootstrapping procedures: Bootstrapping techniques with 5,000 resamples were employed to test mediation effects in the proposed models. This approach provides robust estimates even when data distributions deviate from normality, enhancing the reliability of results.

Power analysis: sample sizes were determined using Monte Carlo simulations (Schoemann et al., 2017) to ensure adequate power (0.80) for detecting medium effect sizes (*r* = 0.30). These simulations indicated a minimum sample size of 153 participants per study.

Variable treatment: variables were standardized to facilitate interpretation of results. Control variables, including age and gender, were included in all analyses. Study-specific controls, such as the duration of incarceration and whether the crime was violent or non-violent (Study 1A), as well as intervention length (Study 2), were incorporated where appropriate.

Analysis strategy: for Studies 1A and 1B, Hayes (2018) PROCESS Macro (Model 4) was used to test mediation models examining the role of deviant associations in linking the search for meaning to criminal attitudes. For Study 2, changes in key variables (search for meaning, deviant associations, and criminal attitudes) were analyzed using pre-post difference scores, followed by mediation testing using bootstrapped estimates.

## Studies 1A-1B

Study 1A was conducted in Pakistan’s correctional services, which house over 70,000 inmates. As a developing nation, Pakistan faces significant socio-economic challenges, contributing to pervasive crime, particularly in urban hubs. This study provided a unique opportunity to examine criminal attitudes among former inmates no longer incarcerated. Participants from the Khyber Pakhtunkhwa region provided informed consent after receiving comprehensive details about the study’s purpose, risks, and benefits.

Study 1B focused on youth from La Línea de la Concepción in Spain’s Campo de Gibraltar region, a high-risk but non-offending population. This area, with 62,940 inhabitants (Instituto de Estadística y Cartografía de Andalucía, 2018), faces rising crime due to factors like its proximity to Gibraltar and Morocco—key routes for smuggling tobacco and hashish. Additionally, the region suffers from high unemployment (over 30% since 2007, peaking at 70% among youth in some neighborhoods), low education levels (20% lacking formal education), and urban structures that concentrate social housing near vulnerable areas (Ayuntamiento de La Línea de la Concepción, 2016). These socio-economic conditions have made local youth prime targets for recruitment by criminal networks, which exploit limited opportunities by promising social and economic advancement. This has fostered a positive perception of these networks in marginalized communities, complicating authorities’ efforts to curb their influence. As a result, the region has experienced increased violence, including attacks on public institutions and officials.

### Participants

The sample of Study 1A included 243 former inmates in Pakistan (52 women; *M*_age_ = 23.58 years, *SD*_age_ = 6.18). They had spent an average of 21.80 months in prisons (*SD* = 12.47), were arrested for various crimes (12.3% drug possession; 74.9% stealing; 12.8% kidnapping), and were currently on parole. The sample of Study 1B included 402 high school students in La Línea de la Concepción, Spain (182 women; *M*_age_ = 14.00 years, *SD*_age_ = 1.41, age range: 12–18); parental consent was obtained and participants completed the survey in a classroom.

### Procedure and materials

#### Search for meaning

Two items taken from Steger et al.’s (2006) meaning in life questionnaire were used to measure participants’ search for meaning (i.e., “I am looking for something that makes my life feel meaningful” and “I am seeking a purpose or mission for my life”; Study 1A: *M* = 6.62, *SD* = 0.54; both items were correlated, *r_s_* = 0.29, *p* < 0.001). A third item was added in Study 1B (i.e., “I am always looking to find my life’s purpose” *M* = 4.81, *SD* = 1.59, *α* = 0.77). Participants provided their responses on a 7-point Likert scale ranging from 1 (*Not agree at all*) to 7 (*Very strongly agree*).

#### Deviant associations

In Study 1A, we utilized two items adapted from Moyano et al.’s. (under review) scale to measure participants’ affiliation with a criminal network of peers (i.e., “People around me say it is appropriate to use crime to make a living” and “I personally know someone that engages in crime to make a living”; *M* = 5.66, *SD* = 1.87, both items were correlated, *r_s_* = 0.98, *p* < 0.001). In Study 1B, participants completed the full scale which includes eight items (*M* = 2.23, *SD* = 1.21, *α* = 0.82). Participants provided their responses on a 7-point Likert scale ranging from 1 (*Not agree at all*) to 7 (*Very strongly agree*).

#### Criminal attitudes

In Study 1A, participants’ support for criminal activities was measured using six items (e.g., “It is acceptable to participate in illegal activities to make a living” and “There are effective ways of earning a living other than using crime” reverse-score; *M* = 5.72, *SD* = 1.02, *α* = 0.66). In Study 1B, participants’ support for criminal activities was measured using three items (*M* = 2.95, *SD* = 1.72, *α* = 0.66). Participants provided their responses on a 7-point Likert scale ranging from 1 (*Not agree at all*) to 7 (*Very strongly agree*).

#### Control variables

In Studies 1A-1B, age and gender were included as control variables. In Study 1A, we additionally controlled for the amount of time participants had spent in prison and the “Type of Crime” variable was coded as dichotomous (1 = violent, 0 = non-violent) to facilitate analysis of its relationship with other study variables. The mean of this variable (*M* = 0.61, *SD* = 0.48) reflects the proportion of participants involved in violent crimes, with 61% of the sample categorized as violent offenders and the remaining 39% as non-violent offenders. Violent crimes encompassed offenses such as robbery, kidnapping, and mobile snatching, characterized by the use or threat of force against individuals. Non-violent crimes included drug possession, pickpocketing, and theft, with the latter two involving the unlawful acquisition of property without direct physical confrontation.

## Results

**Figure 1:**
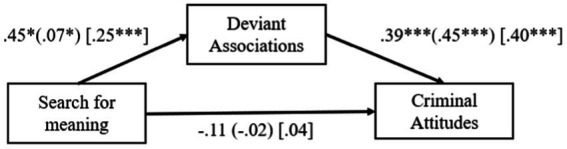
Unstandardized beta coefficients for the relationship between search for meaning, deviant associations, and criminal attitudes (Studies 1A-1B, 2). *p < 0.05, ***p < 0.001. Estimates for Studies 1A and 1B are presented outside and inside parentheses, respectively, while estimates for Study 2 are enclosed in brackets.

We examined whether deviant associations mediated the relationship between search for meaning and criminal attitudes (H1). Table 1 shows the means and standard deviations, and correlations for all measures. Results indicated that search for meaning was related to deviant associations (Study 1A: *B* = 0.45, *SE* = 0.22, *p* = 0.04; Study 1B: *B* = 0.07, *SE* = 0.03 *p* = 0.03), which in turn was associated with criminal attitudes (Study 1A: *B* = 0.39, *SE* = 0.02, *p* < 0.001; Study 1B: *B* = 0.45, *SE* = 0.06, *p* < 0.001; see Figure 1). The model explained 54 and 13% of the variance in criminal attitudes for Studies 1A and 1B, respectively.

**Table 1:** Means, standard deviations, and correlations for all measures of Studies 1A-1B.

	*M*	*SD*	1	2	3	4	5	6
1.Search for meaning	6.62(4.81)	0.54(1.59)						
2.Deviant associations	5.66(2.23)	1.87(1.21)	0.11^†^(0.10*)					
3.Criminal attitudes	5.72(2.95)	1.02(1.72)	0.02(0.01)	0.72***(0.33***)				
4.Age	23.58(14.00)	6.18(0.49)	−0.01(0.03)	0.03(0.10*)	0.13*(0.12*)			
5.Gender	0.21(1.45)	0.41(0.49)	0.14*(0.03)	−0.05(−0.06)	−0.05(−0.15**)	0.01(0.02)		
6.Months in Prison	21.80	12.47	0.05	−0.06	−0.01	0.02	−0.14*	
7.Type of Crime	0.61	0.48	−0.06	0.03	0.09	0.01	−0.61***	0.16**

^†^*p* < 0.07, *p < 0.05, ***p < 0.001. Estimates for Studies 1A and 1B are outside and inside the parentheses, respectively.

Estimates of the indirect effect showed that the relationship between search for meaning and supporting criminal activities was mediated by deviant associations (Study 1A: *B* = 0.18, *SE* = 0.17, 95% CI = [0.01, 0.36]); (Study 1B: *B* = 0.03, *SE* = 0.01, 95% CI = [0.001, 0.07]). Notably, in Study 1A, none of the control variables showed a significant relationship with deviant associations (all *p*s > 0.20). However, age was related to criminal attitudes (*B* = 0.01, *SE* = 0.007, *p* = 0.01; all other *p*s > 0.11). Of note, having committed a violent crime correlated negatively with being a woman (*r* = −0.61, *p* < 0.001) and positively with length of sentence (*r* = 0.16, *p* < 0.01), suggesting that women were less likely to commit violent crimes, which were associated with longer sentences. In Study 1B, age (*B* = 0.08, *SE* = 0.04, *p* = 0.04) was related to deviant associations, but not gender (*p* = 0.12). Criminal attitudes were significantly associated with both age (*B* = 0.11, *SE* = 0.05, *p* = 0.04) and gender (coded 1 for female, 0 for male; *B* = − 0.45, *SE* = 0.16, *p* = 0.005). Specifically, older teenagers were more likely to have a positive attitude toward criminal behavior, while males were more prone to such attitudes compared to females.

The results of our initial test of the 3N model showed a significant relationship between search for meaning, deviant associations, and criminal attitudes. Notably, deviant associations were found to mediate the connection between search for meaning and criminal attitudes, explaining a substantial portion of the variance in both Study 1A and Study 1B. Surprisingly, traditional criminogenic variables, such as crime type and time spent in prison, did not show significant associations with criminal attitudes, suggesting that deviant associations play a more prominent role in influencing individuals’ criminal attitudes.

## Study 2

Study 2 utilized data from the Fénix Andalucía project, an initiative by the Andalusian government based on the 3N model, aimed at fostering social inclusion in underprivileged areas. Targeting students in their final years of primary and lower secondary education, the program hypothesized that fostering pro-social associations could redirect the search for meaning away from criminal attitudes. Activities included participative learning and mentoring sessions led by trained teachers and counselors.

Students developed competencies in artistic, scientific, communicative, and entrepreneurial domains. Schools were assigned mentors, such as sports figures, musicians, artists, or individuals with inspiring self-improvement stories, who served as role models. Local role models also delivered talks and videos sharing personal experiences. The intervention lasted 6–8 weeks per school.

Effectiveness was evaluated through pre-post assessments of 3N model factors, focusing on data from 23 disadvantaged schools scoring 3 or below on the Economic, Social, and Cultural Status Index (ESCS). This index integrates information about students’ social and family contexts, including parental education, occupation, household income, access to resources, and availability of books at home (Ineval, 2017).

### Participants

The sample for this study comprised 330 students (176 women; *M*_age_ = 12.03 years, *SD*_age_ = 1.27) from 23 primary and secondary schools located in the most deprived areas of the Andalusia (Spain) region. According to the Spanish educational system, 59 participants (18%) were in fifth grade of primary education, 116 (35%) in sixth grade of primary education, 94 (29%) in the first year of secondary education (ESO), and 61 (18%) in the second year of secondary education (ESO). Prior to data collection, parental consent was obtained. Given the youth of the participants, the questionnaire was worded to align with their cognitive capabilities. That is, terms beyond their comprehension (e.g., meaning in life) were avoided, as well as explicit references to crimes or other unlawful acts.

### Procedure and materials

#### Search for meaning

We included four items related to self-esteem to assess the search for meaning, aligning with the 3N theory, which suggests that self-esteem contributes significantly to individuals’ perceptions of existential purpose and significance. The items were taken from Álvarez-Ramírez (2012): “I feel that I am as valuable a person as the others” (reverse-scored), “I am generally inclined to think of myself as a failure,” “Sometimes I think I am good for nothing,” and “I often feel lonely when I am with other people.” Participants rated these items on a 5-point Likert scale ranging from 1 (*Not agree at all*) to 5 (*Completely agree*) (T1: *M* = 2.20, *SD* = 0.96, *α* = 0.70; T2: *M* = 2.06, *SD* = 0.91, *α* = 0.67). To calculate an index reflecting the change in this variable, we subtracted the T1 scores from the T2 scores (*M* = −0.13, *SD* = 0.94).

#### Deviant associations

We included three items from Moyano’s deviant associations scale (2011) to measure participants’ association with deviant peers (i.e., “My friends talk about fights and violence all the time”), “My friends get into too much trouble (stealing, drugs, fighting, etc.,” and “Some people tell me that doing illegal activities is useful to make a living”). Participants rated these items on a 5-point Likert scale, ranging from1 (*Not agree at all*) to 5 (*Completely agree*) (T1: *M* = 1.95, *SD* = 0.94, *α* = 0.67; T2: *M* = 1.88, *SD* = 0.97, *α* = 0.73). To calculate an index reflecting the change in association with deviant peers, we subtracted the T1 scores from the T2 scores (*M* = −0.06, *SD* = 1.04).

#### Criminal attitudes

For the assessment of criminal attitudes, we utilized four items taken from the scales developed by Bélanger et al. (2019) and Huesmann et al. (2011) (i.e., “If you are angry, it’s okay to say mean things to other people,” “It is okay to push or abuse other people if you are angry,” “It is okay to let off steam with others by using force” and “Violence is necessary for social change”). Participants rated these items on a 5-point Likert scale, ranging from 1 (*Not agree at all*) to 5 (*Completely agree*) (T1: *M* = 1.63, *SD* = 0.84, *α* = 0.80; T2: *M* = 1.51, *SD* = 0.81, *α* = 0.82). To calculate an index reflecting the change in criminal attitudes, we subtracted the T1 scores from the T2 scores (*M* = −0.11, *SD* = 0.97).

In addition to coding and controlling for age and gender, we also factored in the duration of time between T1 and T2 as an indicator of the intervention’s length. As mentioned earlier, this duration varied across schools, with an average of 69.35 days and a standard deviation of 16.46. Correlations between the variables are reported in Table 2.

**Table 2:** Means, standard deviations, and correlations for all measures of Study 2.

	*M*	*SD*	1	2	3	4	5	6	7	8	9	10	11
1.Search for meaning (T1)	2.20	0.96											
2.Search for meaning (T2)	2.06	0.91	0.51^***^										
3.Search for meaning (index)	−0.13	0.93	−0.53^***^	0.45^***^									
4.Deviant associations (T1)	1.95	0.94	0.25^***^	0.10	−0.16^**^								
5.Deviant associations (T2)	1.88	0.97	0.14^*^	0.23^***^	0.09	0.40^***^							
6.Deviant associations (index)	−0.06	1.04	−0.10	0.12^*^	0.23^***^	−0.52^***^	0.57^***^						
7.Criminal attitudes (T1)	1.63	0.84	0.25^***^	0.06	−0.19^***^	0.57^***^	0.21^***^	−0.31^***^					
8.Criminal attitudes (T2)	1.51	0.81	0.17^***^	0.16^**^	−0.02	0.26^***^	0.49^***^	0.22^***^	0.30^***^				
9.Criminal attitudes (index)	−0.11	0.97	−0.07	0.07	0.14^**^	−0.27^***^	0.22^***^	0.45^***^	−0.60^***^	0.57^***^			
10.Age	12.03	1.27	0.02	0.11^*^	0.07	0.04	0.05	0.00	0.08	0.21^***^	0.10^*^		
11.Gender	1.47	0.50	−0.18^***^	−0.07	0.12^*^	0.13^*^	0.15^**^	0.02	0.12^*^	0.14^**^	0.01	−0.005	
12.€Duration of the program	69.35	16.46	0.08	0.12*	0.03	−0.07	0.05	0.12^*^	−0.03	0.09	0.11^*^	0.09	−0.03

*p < 0.05, **p < 0.01, ***p < 0.001.

## Results

We performed Student’s *t*-test for paired samples to test the differences between T1 and T2 scores across the main factors. The results revealed a statistically significant decrease in search for meaning [*t*(329) = 2.72, *p* = 0.007, *d* = 0.14], a non-significant reduction for deviant associations [*t*(329) = 1.20, *p* = 0.22, *d* = 0.07] and a significant reduction in criminal attitudes [*t*(329) = 2.21, *p* = 0.02, *d* = 0.14]. Next, we used the indexes of change for each variable to test the same theoretical model as in Studies 1A-1B, using Hayes (2018) PROCESS Macro, Model 4. Specifically, we examined whether changes in search for meaning would be associated with a decrease in criminal attitudes through a decrease in deviant associations (H2; see Figure 1). Results indicated that changes in the search for meaning was related to changes in deviant associations (*B* = 0.25, *SE* = 0.06, *p* < 0.001), which in turn was associated with changes in criminal attitudes (*B* = 0.40, *SE* = 0.04, *p* < 0.001). The model explained 22% of the variance in criminal attitudes.

Estimates of the indirect effect showed that the relationship between search for meaning and supporting criminal activities was mediated by deviant associations (*B* = 0.10, *SE* = 0.03, 95% CI = [0.03, 0.19]). Regarding the control variables, the duration of the intervention was related to deviant associations (*B* = 0.007, *SE* = 0.003, *p* = 0.02), but not to age (*p* = 0.69) or gender (*p* = 0.98). Furthermore, criminal attitudes were predicted by age (*B* = 0.06, *SE* = 0.03, *p* = 0.06), but not by gender (*p* = 0.94) or the duration of the intervention (*p* = 0.34).

Overall, the results showed that the program led to a decrease in the search for meaning and criminal attitudes, although it did not result in a significant reduction in deviant associations. Consistent with the findings of Studies 1A-1B, results showed that deviant associations (networks) mediate the relationship between the search for meaning (needs) and criminal attitudes (narratives).

## General discussion

The primary objective of this research was to investigate the applicability of the 3N model, originally designed to predict radical attitudes, to the realm of criminal attitudes. By leveraging insights from this model (Bélanger et al., 2020; Lobato et al., 2021), our investigation focused on needs and networks as potential predictors of narratives, which, in the context of this research, were indicative of criminal attitudes. Recognizing the extensive convergence in the scientific literature between radical and criminal attitudes (Wolfowicz et al., 2020), our hypotheses were centered on the premise that criminal attitudes would be predicted in a comparable manner to radical attitudes. Specifically, we hypothesized a positive relationship between the search for meaning and criminal attitudes, mediated by deviant associations (H1). Additionally, we postulated that fostering pro-social associations could redirect the search for meaning away from criminal attitudes (H2).

Our first hypothesis received support from the results of Study 1A, which examined a sample of offenders in Pakistan. The findings indicated a significant indirect effect of the search for meaning on criminal attitudes, mediated through deviant associations. This pattern was replicated in Study 1B with at-risk youth in Spain, where we found no direct relationship between the search for meaning and criminal attitudes; rather, the relationship was mediated by deviant associations. The findings from Studies 1A-1B suggest that radical attitudes and criminal attitudes share similar mechanisms, further strengthening the conceptual overlap between these constructs. Aligning with recent studies that applied the 3N model of radicalization and found similar patterns (e.g., Bélanger et al., 2020; Lobato et al., 2021), our research illustrates the trajectory from the search for meaning to association with deviant peers and, ultimately, to the adoption of violence-supportive beliefs.

In Study 2, we investigated the second hypothesis within underprivileged areas of Spain. The results indicated that the intervention, focusing on positive role models for at-risk youth, effectively reduced both the search for meaning and criminal attitudes. Moreover, we found a significant association between changes in the search for meaning and deviant associations, which, in turn, were linked to criminal attitudes. The findings of this study align with key criminological theories that shed light on the relationship between criminal attitudes and deviant associations. Social learning theory, for instance, suggests that the adoption of criminal attitudes is influenced by the attitudes observed in an individual’s social network, including peers, family, teachers, and role models, acting as sources of imitation (Akers, 1998). Similarly, self-control theories propose that life events, such as experiencing a loss of significance or strains, may trigger sensation-seeking behaviors that lead to the formation or reinforcement of deviant associations (Gottfredson and Hirschi, 1990). While these theories offer valuable insights, they do not explicitly consider meaning in life as a protective factor against developing deviant associations and negative peer influences. Thus, the findings of Study 2 contribute to those ideas by showing that pro-social associations may redirect the search for meaning away from criminal attitudes, thereby disrupting the potential pathway to negative outcomes.

One notable strength of our research lies in its diverse sample composition. Study 1A focused on Pakistani offenders, while Study 1B and Study 2 examined at-risk youth in Spain. This diversity in the samples allows us to explore the applicability of the 3N model across various populations and cultural contexts. The fact that the model can be effectively utilized across these different groups suggests its potential for broader generalizability when studying criminal attitudes. This alignment with the fundamental premise of the 3N model, which posits the search for meaning as a universal human need, enhances the robustness of our findings and reinforces the model’s capacity to illuminate the complex relationship between the search for meaning and criminal attitudes.

### Cultural and developmental variability across studies

The variability in correlations across the studies highlights the importance of cultural, developmental, and contextual factors. Study 1A, conducted among former inmates in Pakistan, showed a weak correlation (*r* = 0.10) between deviant associations and the search for meaning. In a setting marked by systemic socio-economic challenges and high crime rates, deviant associations may be driven more by immediate environmental pressures and social influences than by existential motivations. Additionally, former inmates may have re-evaluated their search for meaning post-incarceration, reducing its connection to deviant associations. Age in this sample showed a small positive correlation with criminal attitudes (*r* = 0.13) but was unrelated to deviant associations or search for meaning. These findings suggest that older individuals in this sample may develop more entrenched criminal attitudes over time, potentially due to the cumulative effect of life experiences or sustained exposure to criminogenic environments. However, this trend appears independent of peer influences (deviant associations) or existential concerns (search for meaning), indicating that criminal attitudes in older individuals may stem more from internalized beliefs or habitual patterns rather than ongoing external or existential factors.

In contrast, Studies 1B and 2 involved at-risk youth in Spain, sharing similar ages but differing in the contextual focus of the research. Study 1B examined youth in La Línea de la Concepción, a region where criminal networks have normalized deviant associations as pathways to economic and social survival. Here, the weak correlation (*r* = 0.11) between deviant associations and the search for meaning suggests that deviant associations are shaped more by practical considerations than by existential exploration. Gender also played a role, with males in this sample showing higher associations with deviant peers and criminal attitudes (*r* = 0.15), reflecting the influence of gender norms in this high-risk context. The high unemployment, low educational attainment, and the influence of smuggling operations in the region further highlight the immediacy of survival-based motives over meaning-seeking processes.

Study 2, part of the Fénix Andalucía project targeting similarly disadvantaged youth, showed slightly higher correlations (*r* = 0.25 and *r* = 0.14) between deviant associations and the search for meaning. Unlike Study 1B, this intervention actively engaged students in activities fostering identity exploration, offering pro-social role models and emphasizing personal growth. These elements may have heightened participants’ awareness of meaning-seeking processes, thus strengthening the connection between deviant associations and the search for meaning compared to Study 1B. Gender in this sample showed a unique pattern: being male was negatively correlated with search for meaning (*r* = −0.18) but positively associated with deviant associations (*r* = 0.13 and *r* = 0.15) and criminal attitudes (*r* = 0.12 and *r* = 0.14). This suggests that male participants in Study 2 were more likely to engage in deviant behaviors and attitudes while being less focused on existential concerns, a pattern distinct from Study 1A.

The correlations between deviant associations and criminal attitudes varied significantly across the studies, ranging from 0.33 in Study 1B to 0.57, 0.49 in Study 2, and 0.72 in Study 1A. The strong correlation in Study 1A reflects the entrenched nature of criminal attitudes among former inmates, shaped by prolonged exposure to deviant networks and environments. Compared to Study 1B, where the weaker correlation (*r* = 0.33) may reflect the more flexible attitudes of youth navigating socio-economic pressures and limited opportunities, participants in Study 1A appear to have developed stronger, more stable links between deviant associations and criminal attitudes due to repeated reinforcement in criminal contexts. Study 2’s moderate correlations (*r* = 0.57 and *r* = 0.49) suggest a transitional stage, where deviant associations increasingly influence criminal attitudes but remain less entrenched than in Study 1A. This progression highlights how age, exposure, and developmental stage affect the strength of these associations across contexts, while control variables like gender provide additional insight into subgroup differences.

In light of these findings, it becomes apparent that the search for meaning should not be considered a criminogenic factor. This is because the search for meaning, being a fundamental human desire, can lead individuals in either direction – toward or away from deviant behavior (Kruglanski et al., 2019). What matters is how this search is directed, and it is evident that social associations play a pivotal role in influencing individuals toward either positive or deviant behaviors. This perspective aligns seamlessly with the principles of “positive criminology” and its related field, “positive psychology” which concentrate on criminogenic factors, but also place a strong emphasis on nurturing positive elements. One prominent example of this perspective is the Good Lives Model (GLM) of offender rehabilitation, which aims to assist offenders in leading fulfilling and pro-social lives by teaching them skills and strategies to achieve these fundamental goods in lawful and positive ways (Harkins et al., 2012).

In summary, the present work highlights the viability of investigating criminal attitudes using a theoretical framework akin to radical attitudes, such as the 3N model of radicalization. By examining the relationship between the search for meaning and criminal attitudes, this research contributes to the field, illuminating an aspect that has been mentioned in the literature but not extensively studied. Furthermore, it elucidates the mechanism through which the search for meaning can foster such attitudes, involving deviant associations. These findings align with prior research on radical attitudes, fostering further integration between criminological and psychological literature.

### Limitations and future research

The present research has certain limitations that warrant consideration. One limitation of Study 1A is that the nature of the data only permits us to speculate that the relationship between search for meaning, deviant associations, and criminal attitudes existed prior to the occurrence of offending behavior. Similarly, in Study 1B, we can only conjecture that the relationship between search for meaning and deviant associations occurred before the development of criminal attitudes. While our research was based on prior empirical work (Bélanger et al., 2020; Lobato et al., 2021), it is essential to acknowledge the possibility of an alternative trajectory. It is plausible that criminal attitudes could lead to greater deviant associations, subsequently triggering a search for meaning. This alternative pathway would also align with the 3N model, which allows for the emergence of needs, narratives, and networks in no specific temporal order (Kruglanski et al., 2019). Longitudinal research would be necessary to gain further clarity on the validity of this proposition. By exploring the temporal sequence of these factors, future studies can provide insights into the underlying mechanisms shaping criminal attitudes.

Another limitation is that within the confines of Study 2’s data, we were restricted to solely exploring immediate and short-term impacts. It is unlikely that a three-month intervention would lead to long-lasting impacts, especially in high-risk environments where multiple risk factors exist. Additionally, like many interventions of this kind, it is challenging to isolate the specific components that contributed to the observed effects. However, based on the results of Studies 1A and 1B, along with support from existing theoretical and empirical evidence, we propose that the intervention played a role in providing meaning in life.

The findings of this research offer insights into potential directions for future investigations, particularly by delving into the sociocognitive mechanisms that may elucidate how the search for meaning influences criminal attitudes beyond the role of deviant associations. One intriguing aspect worth exploring further is the notion of goal-shielding. Goal-shielding refers to the cognitive mechanism wherein the activation of an important goal leads individuals to focus and commit to that specific task or objective, diminishing attention and responsiveness to alternative goals that could interfere with its achievement (Bélanger et al., 2022). In this context, when the search for meaning is activated, individuals may suppress other considerations and become more inclined to consider means that would otherwise be deemed forbidden. This phenomenon raises the possibility that the search for meaning can shape individuals’ moral judgment, potentially leading them to adopt a utilitarian perspective, where “the end justifies the means,” thus contributing to the adoption of criminal attitudes. By honing in on these sociocognitive mechanisms associated with universal drives like the search for meaning, future research has the potential to unveil the complex dynamics underpinning criminal attitudes. This knowledge could pave the way for the development of more effective strategies in intervention and prevention efforts to address the roots of criminal attitudes.

Moreover, future research would benefit from conducting observational research, particularly when implementing prevention programs similar to the one discussed in Study 2. Despite the inherent challenges associated with observational research, closely observing individuals’ behaviors as they undergo different interventions can minimize biases and potential social desirability effects. This approach can provide concrete behavioral information that helps triangulate the effect of the intervention with traditional assessment tools, such as Likert scales. By integrating these diverse research methodologies, future investigations can enhance the validity and comprehensiveness of their findings, leading to a more robust understanding of how the search for meaning influences criminal attitudes and the long-term impact of interventions.

### Prevention and policy implications

The search for meaning, identified here as a protective factor, holds practical promise due to its universal nature, as all individuals naturally seek meaning in life (Frankl, 1997). Notably, Study 2’s findings emphasize the crucial role of positive role models in supporting adolescents, offering guidance and positive examples that facilitate the development of life goals and a sense of purpose. Consequently, nurturing positive role models may play a pivotal role in reducing the appeal of engaging in criminal activities among young individuals.

These implications carry policy significance, including investing in training and professional development for individuals working with adolescents, such as teachers, social workers, and community leaders, to emphasize the importance of positive role models and effective mentorship for young people. Collaborating with community leaders and organizations to promote positive role models and mentorship opportunities within the community could be instrumental in fostering a culture of positive influence and support for adolescents. Additionally, supporting research and evaluation efforts to understand the effectiveness of various strategies in providing positive role models and mentorship to adolescents can inform the development of evidence-based policies and programs aimed at preventing criminal attitudes and behaviors.

## Conclusion

In high-risk environments, criminality can become enticing to individuals seeking meaning when conventional or pro-social means seem unattainable. The connection between this search for meaning and criminal attitudes is mediated by deviant associations. Understanding these dynamics can significantly enhance primary and secondary prevention strategies. Despite being underexplored in criminological literature, the impact of the search for meaning on both pro-social and criminal attitudes makes it a valuable target for interventions. Approaches grounded in positive criminology and psychology hold promise in redirecting this search for meaning and curbing criminal behavior. Further research in this area has the potential to lead to effective preventive measures.

## Data Availability

The raw data supporting the conclusions of this article will be made available by the authors, without undue reservation.

## Footnotes

^1^Radical or extremist attitudes encompass various ideologies, each with distinct specifics. For instance, right-wing extremism may involve the rejection of liberal, democratic, and egalitarian social norms, while left-wing extremism may reject capitalism and social hierarchies. However, it’s important to note that these attitudes, in and of themselves, do not necessarily advocate for or justify the use of violent means to achieve their goals. In the realm of radicalization research, defining and measuring radical attitudes has sparked extensive debate. While a consensus has yet to be reached, the description provided above broadly reflects the approaches taken by most researchers.
