# Supplementary material for: Beyond radicalization: the 3N model and its application to criminal attitudes in high-risk contexts
Source: Front Psychol. 2025 Feb 17;16:1498936. doi: 10.3389/fpsyg.2025.1498936 (PMC11875101; doi:10.3389/fpsyg.2025.1498936)
Supplement: Supplementary file 1 [file Data_Sheet_1.PDF]

Supplementary File

Study 1A – Pakistan

a) Search for Meaning

1. Not agree at all
2. Very slightly agree
3. Slightly agree
4. Moderately agree
5. Mostly agree
6. Strongly agree
7. Very Strongly agree

a) I am looking for something that makes my life feel meaningful.
b) I am seeking a purpose or mission for my life.

b) Deviant Associations

1. Not agree at all
2. Very slightly agree
3. Slightly agree
4. Moderately agree
5. Mostly agree
6. Strongly agree
7. Very Strongly agree

a) People around me say it is appropriate to use crime to make a living.
b) I personally know someone that engages in crime to make a living.

c) Criminal Attitudes

1. Not agree at all
2. Very slightly agree
3. Slightly agree
4. Moderately agree
5. Mostly agree
6. Strongly agree
7. Very Strongly agree

a) When using crime to earn a living, anybody can be a target.
b) Violence is necessary to make a living.
c) It is acceptable to engage in crime to make a living.
d) I would never consider crime to earn a living.
e) We should never use crime to earn a living.
f) There are effective ways of earning a living other than using crime.

Study 1B – Spain

a) Search for Meaning

1. Not agree at all
2. Very slightly agree
3. Slightly agree
4. Moderately agree
5. Mostly agree
6. Strongly agree
7. Very Strongly agree

a) I am looking for something that makes my life feel meaningful.
b) I am seeking a purpose or mission for my life.
c) I am always looking to find my life's purpose.

b) Deviant Associations

1. Not agree at all
2. Very slightly agree
3. Slightly agree
4. Moderately agree
5. Mostly agree
6. Strongly agree
7. Very Strongly agree

a) My friends talk about fighting and violence all the time.
b) I have several friends or relatives who are or have been in trouble with the law at some point in their lives.
c) My friends get into too much trouble (stealing, drugs, fights, etc.).
d) Some people tell me that doing illegal activities is useful for earning a living.
e) I have sometimes been criticised for not engaging in illegal activities.
f) People around me say it is appropriate to do illegal activities for a living.
g) The people I know would do illegal activities for a living
h) I personally know someone who does illegal activities for a living.

c) Criminal Attitudes

1. Not agree at all
2. Very slightly agree
3. Slightly agree
4. Moderately agree
5. Mostly agree
6. Strongly agree
7. Very Strongly agree

a) It is acceptable to engage in illegal activities to make a living.
b) I would never participate in illegal activities to earn a living.
c) You should never engage in illegal activities to earn a living.

Study 2 - Spain

a) Search for Meaning

1. Not agree at all
2. Slightly agree
3. Moderately agree
4. Mostly agree
5. Completely agree

a) I feel that I am as valuable a person as the others.
b) I am generally inclined to think of myself as a failure.
c) Sometimes I think I am good for nothing.
d) I often feel lonely when I am with other people.

b) Deviant Associations

1. Not agree at all
2. Slightly agree
3. Moderately agree
4. Mostly agree
5. Completely agree

a) My friends talk about fights and violence all the time.
b) My friends get into too much trouble (stealing, drugs, fighting, etc.
c) Some people tell me that doing illegal activities is useful to make a living.

c) Criminal Attitudes

1. Not agree at all
2. Slightly agree
3. Moderately agree
4. Mostly agree
5. Completely agree

a) If you are angry, it's okay to say mean things to other people.
b) It is okay to push or abuse other people if you are angry.
c) It is okay to let off steam with others by using force.
d) Violence is necessary for social change.
